# Identification of nodal micrometastasis in colorectal cancer using deep learning on annotation-free whole-slide images

**DOI:** 10.1038/s41379-021-00838-2

**Published:** 2021-06-08

**Authors:** Wen-Yu Chuang, Chi-Chung Chen, Wei-Hsiang Yu, Chi-Ju Yeh, Shang-Hung Chang, Shir-Hwa Ueng, Tong-Hong Wang, Chuen Hsueh, Chang-Fu Kuo, Chao-Yuan Yeh

**Affiliations:** 1 Department of Pathology, Chang Gung Memorial Hospital and Chang Gung University, Taoyuan, Taiwan; 2 aetherAI Co., Ltd., Taipei, Taiwan; 3 Center for Big Data Analytics and Statistics, Chang Gung Memorial Hospital, Taoyuan, Taiwan; 4 Chang Gung Molecular Medicine Research Center, Chang Gung University, Taoyuan, Taiwan; 5 Tissue Bank, Chang Gung Memorial Hospital, Taoyuan, Taiwan; 6 Center for Artificial Intelligence in Medicine, Chang Gung Memorial Hospital, Taoyuan, Taiwan

**Keywords:** Colorectal cancer, Metastasis

## Abstract

Detection of nodal micrometastasis (tumor size: 0.2–2.0 mm) is challenging for pathologists due to the small size of metastatic foci. Since lymph nodes with micrometastasis are counted as positive nodes, detecting micrometastasis is crucial for accurate pathologic staging of colorectal cancer. Previously, deep learning algorithms developed with manually annotated images performed well in identifying micrometastasis of breast cancer in sentinel lymph nodes. However, the process of manual annotation is labor intensive and time consuming. Multiple instance learning was later used to identify metastatic breast cancer without manual annotation, but its performance appears worse in detecting micrometastasis. Here, we developed a deep learning model using whole-slide images of regional lymph nodes of colorectal cancer with only a slide-level label (either a positive or negative slide). The training, validation, and testing sets included 1963, 219, and 1000 slides, respectively. A supercomputer TAIWANIA 2 was used to train a deep learning model to identify metastasis. At slide level, our algorithm performed well in identifying both macrometastasis (tumor size > 2.0 mm) and micrometastasis with an area under the receiver operating characteristics curve (AUC) of 0.9993 and 0.9956, respectively. Since most of our slides had more than one lymph node, we then tested the performance of our algorithm on 538 single-lymph node images randomly cropped from the testing set. At single-lymph node level, our algorithm maintained good performance in identifying macrometastasis and micrometastasis with an AUC of 0.9944 and 0.9476, respectively. Visualization using class activation mapping confirmed that our model identified nodal metastasis based on areas of tumor cells. Our results demonstrate for the first time that micrometastasis could be detected by deep learning on whole-slide images without manual annotation.

## Introduction

For cancer patients, pathologic staging is crucial for choosing a proper treatment strategy. Traditionally, detection of nodal metastasis relies on microscopic examination of all resected lymph nodes by a pathologist. Overlooking a small metastatic focus in a lymph node could result in inaccurate staging and subsequent undertreatment of a patient. Therefore, an assisting tool to detect small metastatic foci in lymph nodes, if available, would be helpful for pathologic staging.

In colorectal cancer, nodal micrometastasis has been defined as a metastatic focus with a size between 0.2 and 2.0 mm by the International Union Against Cancer since 2002 [1], and metastatic foci smaller than 0.2 mm are considered isolated tumor cells. Since a recent meta-analysis showed that micrometastasis is a significant poor prognostic factor [2], a lymph node with micrometastasis is considered a standard positive node in the 8th edition of AJCC Cancer Staging Manual in 2017 [3]. In contrast, despite a minor adverse prognostic effect in a subset of early colorectal cancer patients [4], a lymph node with isolated tumor cells is regarded as a negative node in the AJCC staging [3].

Previously, deep learning algorithms developed from manually annotated images were demonstrated to identify metastatic breast cancer in sentinel lymph nodes, including micrometastasis [5]. Using such algorithms as an assisting tool for pathologists was found to effectively increase the sensitivity and efficiency of detecting micrometastasis [6]. However, their deep learning method requires a large dataset of manually annotated microscopic images, and detailed marking of tumor areas by pathologists is extremely labor intensive and time consuming. Arguably, the requirement of manual annotation has been a major obstacle to the development of artificial intelligence (AI) applications in pathology.

Later on, a method called multiple instance learning (MIL) was used to perform deep learning on whole-slide images (WSIs) without manual annotation [7]. Such an approach was proved useful in detecting prostate cancer in needle biopsies, basal cell carcinoma in skin biopsies, and metastatic breast cancer in axillary lymph nodes [7]. Of note, in their 23 false negative cases of metastatic breast cancer, 13 (56.5%) of them were micrometastasis and 2 were isolated tumor cells. Although the performance of their algorithm in detecting micrometastasis was not described, it appears worse in detecting micrometastasis since micrometastasis is much less common than macrometastasis.

Recently, we developed a new method for training neural networks on undivided WSIs using only a slide-level label [8]. Unlike the MIL method using a two-step approach including patch selection, our new method is using entire WSIs for direct end-to-end training. Since our new method outperformed MIL in subclassification of lung cancer [8], it would be interesting to know if we could train a deep learning model using our new method to detect micrometastasis.

In this study, we developed a deep learning algorithm to detect nodal metastasis of colorectal cancer using our new method of end-to-end training with annotation-free WSIs. The performance of this algorithm in detecting macrometastasis, micrometastasis, and isolated tumor cells was evaluated. The performance on different histologic subtypes of cancer was also analyzed.

## Materials and methods

### Datasets

A total of 3182 slides of regional lymph nodes of colorectal cancer were retrieved from the archives of Department of Pathology, Chang Gung Memorial Hospital in Linkou, Taiwan. These slides were from 1051 patients within a period of 5 years. All slides were routine sections of formalin-fixed paraffin embedded tissue with hematoxylin and eosin (H&E) stain. Whole-slide high-resolution digital images were produced using a NanoZoomer S360 digital slide scanner (Hamamatsu Photonics, Hamamatsu, Japan) with a 40× objective mode. The average size of a WSI was 111,198 × 86,483 pixels (maximum size: 207,360 × 108,288 pixels). This study had been approved by the Institutional Review Board of Chang Gung Memorial Hospital (IRB Nos 201701560B0 and 201800413B0).

Among the 3182 slides, 1589 of them with metastatic cancer cells (either macrometastasis, micrometastasis, or isolated tumor cells) were labeled as “positive slide,” whereas other 1593 slides without cancer cells were labeled as “negative slide.” Two senior pathologists (W-YC and C-JY) reviewed and labeled all slides accordingly. Four slides originally diagnosed as negative were corrected to micrometastasis or isolated tumor cells. No detailed manual annotation was performed. The WSIs were randomly split into training (1963 slides, including 973 positive and 990 negative ones), validation (219 slides, including 116 positive and 103 negative ones), and testing (1000 slides, including 500 each of positive and negative ones) datasets using nonstratified sampling for model training, tuning, and performance evaluation, respectively.

Since most of our slides had more than one lymph node, we also evaluated the performance of our algorithm on images of single-lymph nodes. A single-lymph node testing set (538 single-lymph nodes, including 101 positive and 437 negative ones) was prepared by random selection of WSIs and manual cropping of single-lymph nodes using a free-hand contouring tool on aetherSlide Digital Pathology System (aetherAI, Taipei, Taiwan).

To compare the performance of our algorithm on metastases of different size and histologic subtypes, positive WSIs and single-lymph node images in the slide-level and lymph node-level testing sets were further evaluated by two senior pathologists (W-YC and C-JY). Among the 500 positive WSIs in the slide-level testing set, 439 were macrometastasis, 56 were micrometastasis, and 5 were isolated tumor cells. Among the 101 positive nodes in lymph node-level testing set, 71 were macrometastasis, 25 were micrometastasis, and 5 were isolated tumor cells. Regarding the histologic subtypes of the 500 positive WSIs, 469 were classical adenocarcinoma, 13 were mucinous adenocarcinoma, and 18 were signet ring cell/poorly differentiated adenocarcinoma. Among the 101 positive single-lymph nodes, 74 were classical adenocarcinoma, 14 were mucinous adenocarcinoma, and 13 were signet ring cell/poorly differentiated adenocarcinoma.

### Computer hardware and software

We conducted our experiments on TAIWANIA 2, a multi-graphics processing unit (GPU), multinode supercomputer. It consists of 252 computing nodes, and each node is equipped with eight Tesla V100 32GB-HBM2 GPUs. The software stack was CUDA 10.0 and cuDNN 7.6 for GPU acceleration, TensorFlow 1.15.3 for model building and training, and Open MPI 4.0.1, MPI for Python 3.0.3 and Horovod 0.19.0 for multi-GPU training. The training task was performed with a batch size of eight, one training sample per GPU.

### Whole-slide model training

We trained a ResNet-50 [9] convolutional neural network (CNN) using a whole-slide training pipeline [8] to identify whether an image contains metastasis. The pipeline consisted of data preparation and model update.

For data preparation, a WSI was randomly selected as well as its corresponding slide-level label (positive or negative). The image was resized from 40× to 4× to improve training efficiency. The effective spatial resolution was 22.5 µm/pixel. It was then padded to 21,500 × 21,500 pixels with white color to standardize the image size. The rescaled image was processed by a sequence of data augmentation procedures, including random flipping, random rotation (0°–360°), random translation (±500 pixels in both horizontal and vertical dimensions), random contrast (scaling contrast by 0.5–1.5), random brightness (scaling brightness by 0.65–1.35), random hue (±32), and random saturation (±32). The increased diversity of training images is known to effectively make the model more generalizable and robust [10].

For model update, we trained a ResNet-50 CNN [9] through the whole-slide training method as previously described [8]. The underlying model ResNet-50 was slightly modified, with the batch normalization layers frozen to increase multi-GPU efficiency. Binary cross entropy was adopted as the loss function for model training and evaluation. The model was initialized using ImageNet pretrained weights and updated by an Adam optimizer [11] with an initial learning rate of 0.00001. Along the training process, the model iteratively learned from newly fed augmented images, and was evaluated by calculating the loss of the validation dataset every 88 iterations (i.e., an epoch). When no improvement was made over the last 24 epochs, the learning rate was decreased to 0.000001 for the first time, and the training was stopped for the second time. Only the weights achieving the best validation performance were saved.

### Visualization using class activation mapping (CAM)

We used CAM [12] to highlight relatively important regions of the image for model prediction. Specifically, the CAM method applied its operations on the feature map generated by the last dense layer before the global average pooling layer. The resulting two-dimensional map was then upsampled to the size of the WSI using bicubic interpolation. Areas with higher values in the CAM are more important for identification of metastasis.

### Statistics

We used area under the receiver operating characteristic curve (area under the ROC curve; AUC) as the metric to evaluate the classifier. The 95% confidence intervals (CIs) were calculated using DeLong’s method [13]. For significance testing, we adopted a dummy model that always outputs 0.5 as the prediction result for any WSI as the null hypothesis. The 95% CIs of accuracies were modeled as binomial proportion CIs and calculated through Wilson’s method.

## Results

### Model performance on WSIs

**Fig. 1:**
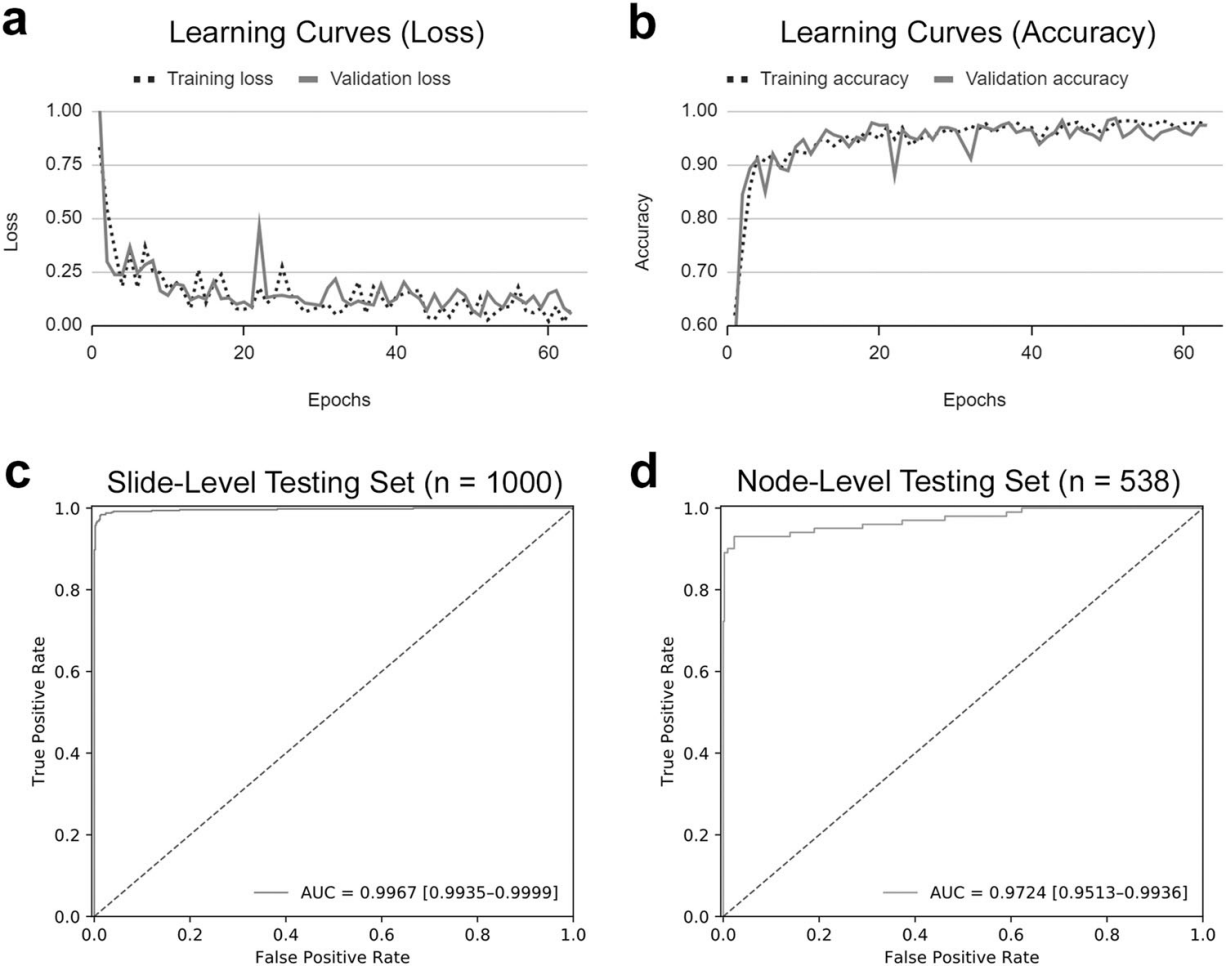
Learning curves and ROC curves of our model. The learning curves of our model showed gradual decrease of loss (**a**) and increase of accuracy (**b**) during the training process. The ROC curves of our model in slide-level testing set (**c**) and single-lymph node-level testing set (**d**).

The learning curves of our model are shown in Fig. 1a, b. The ROC curve of our model on slide-level testing set is shown in Fig. 1c. Our algorithm performed well in identifying metastatic colorectal cancer on the 1000 WSIs of the testing dataset, with an AUC of 0.9957 (95% CI: 0.9935–0.9999). The algorithm achieved an accuracy of 98.50% (95% CI: 97.75%–99.25%) with a prediction threshold of 0.5.

### Model performance on single-lymph node images

The ROC curve of our model on single-lymph node testing set is shown in Fig. 1d. Our algorithm maintained high performance in identifying metastatic colorectal cancer on the 538 single-lymph node images of the testing dataset, with an AUC of 0.9724 (95% CI: 0.9513–0.9936). The algorithm achieved an accuracy of 97.58% (95% CI: 96.29%–98.88%) using a prediction threshold of 0.3.

### Model performance regarding different lesion size

**Fig. 2:**
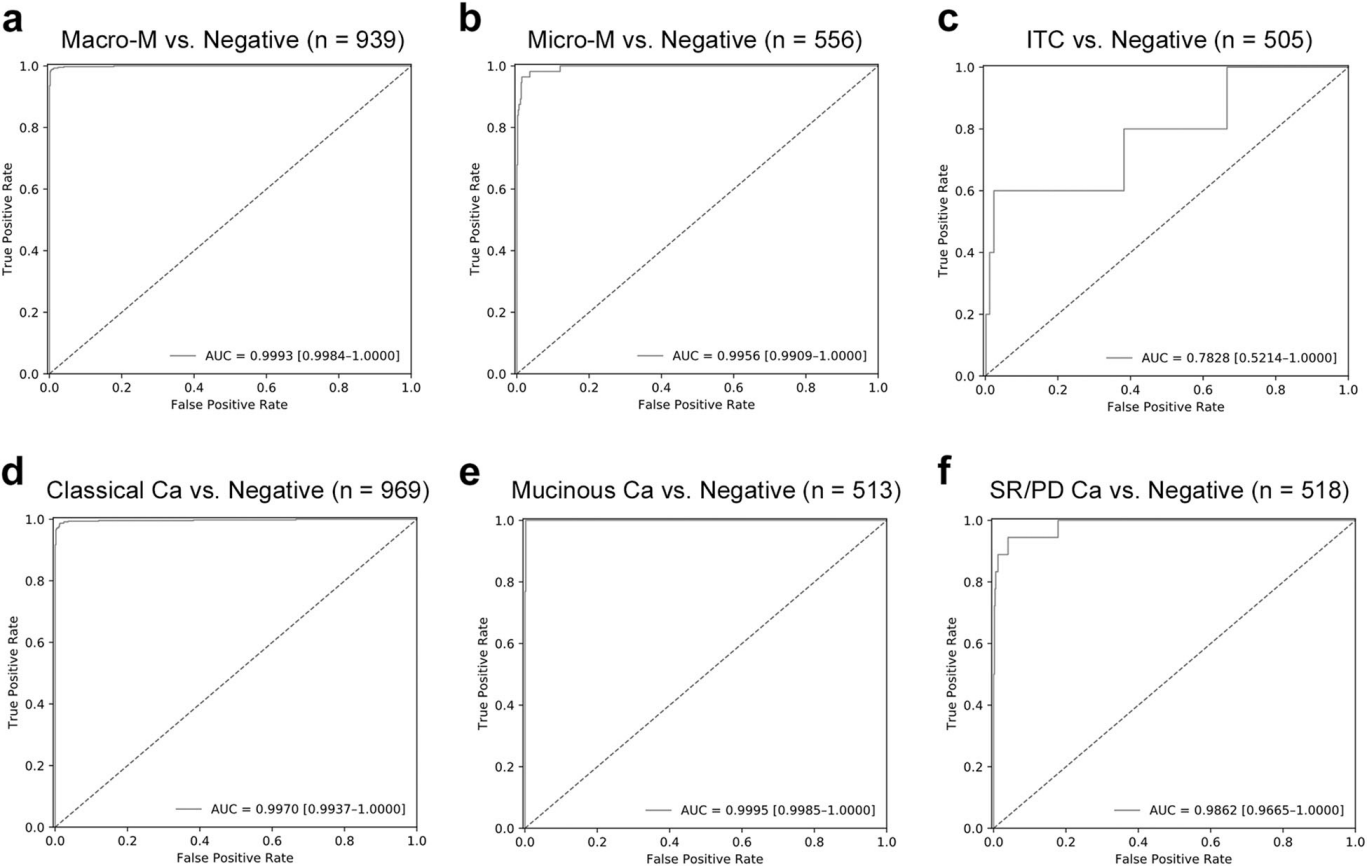
Slide-level ROC curves of our model in detecting different sizes and histologic subtypes of metastasis. The ROC curves of our model in slide-level testing set, including detection of macrometastasis (**a**), micrometastasis (**b**), isolated tumor cells (**c**), classical adenocarcinoma (**d**), mucinous adenocarcinoma (**e**), and signet ring cell/poorly differentiated adenocarcinoma (**f**).

The ROC curves of our model in identifying macrometastasis, micrometastasis, and isolated tumor cells in the slide-level testing set are shown in Fig. 2a–c. The algorithm performed well in identifying macrometastasis and micrometastasis, with an AUC of 0.9993 and 0.9956, respectively. The performance was obviously worse in detecting isolated tumor cells, with an AUC of 0.7828.

**Fig. 3:**
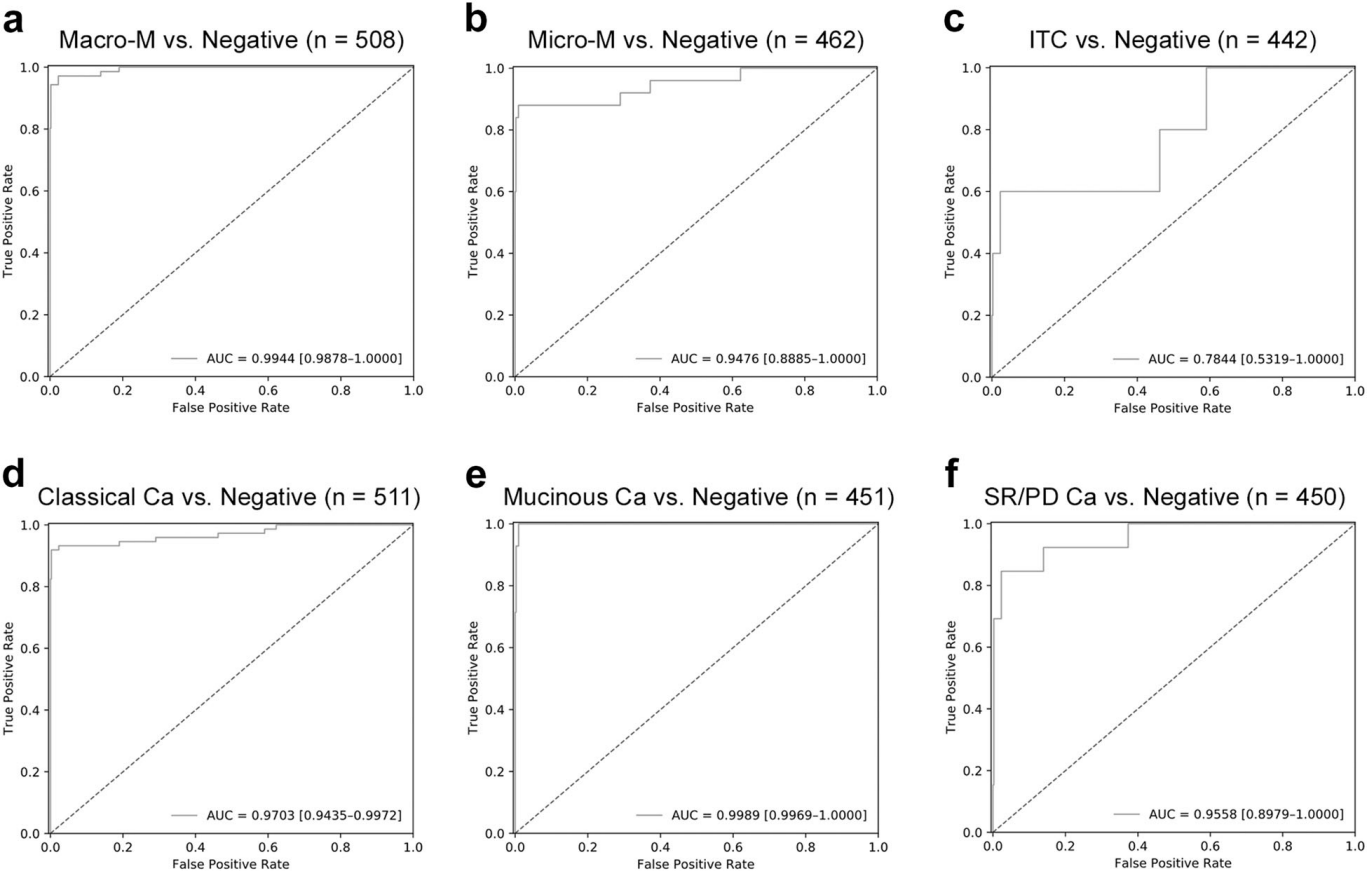
Single-lymph node-level ROC curves of our model in detecting different size and histologic subtypes of metastasis. The ROC curves of our model in single-lymph node-level testing set, including detection of macrometastasis (**a**), micrometastasis (**b**), isolated tumor cells (**c**), classical adenocarcinoma (**d**), mucinous adenocarcinoma (**e**), and signet ring cell/poorly differentiated adenocarcinoma (**f**).

For the single-lymph node-level testing set, the ROC curves are shown in Fig. 3a–c. Similar to the slide-level testing set, the performance was good for macrometastasis and micrometastasis, with an AUC of 0.9944 and 0.9476, respectively. The model achieved lower performance for detection of isolated tumor cells, with an AUC of 0.7844.

### Model performance regarding different histologic subtypes

The ROC curves of our model in identifying classical adenocarcinoma, mucinous adenocarcinoma, and signet ring cell/poorly differentiated adenocarcinoma in the slide-level testing set are shown in Fig. 2d–f. The model performed well in identifying classical adenocarcinoma and mucinous adenocarcinoma, with an AUC of 0.9970 and 0.9995, respectively. The performance was slightly worse in detecting signet ring cell/poorly differentiated adenocarcinoma, with an AUC of 0.9862.

For the single-lymph node-level testing set, the ROC curves are shown in Fig. 3d–f. Similar to the slide-level testing set, the performance was good for classical adenocarcinoma and mucinous adenocarcinoma, with an AUC of 0.9703 and 0.9989, respectively. Detection of signet ring cell/poorly differentiated adenocarcinoma was also slightly worse, with an AUC of 0.9558.

### Key morphologic features for identification of metastatic cancer

**Fig. 4:**
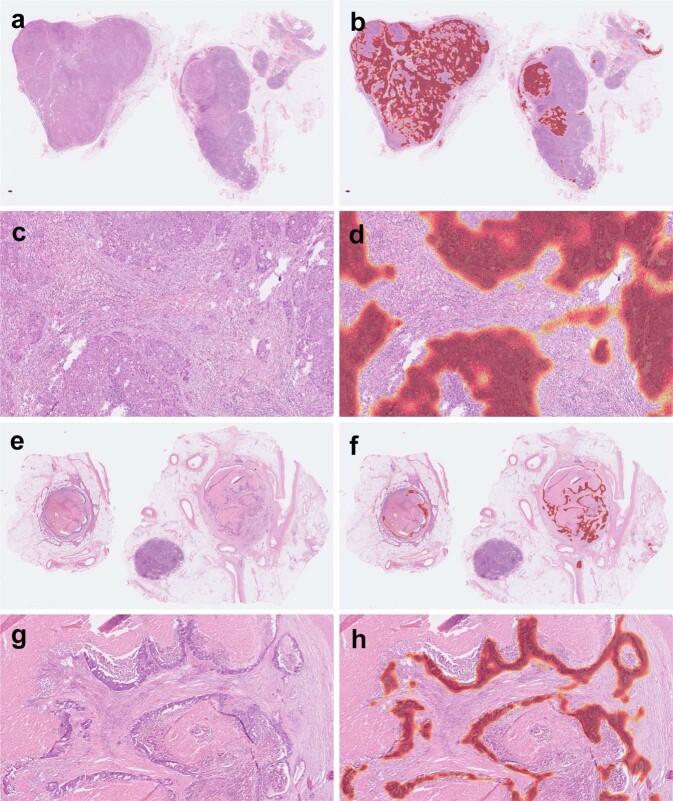
Examples of classical adenocarcinoma in our slide-level testing set. Using class activation mapping, areas of the original whole-slide image (**a**) relatively important for positive prediction were highlighted with red color (**b**). A close-up view (**c**) and its highlighted areas (**d**) showed that our algorithm identified metastasis mainly based on areas of tumor cells. Another example with extensive tumor necrosis demonstrated that our algorithm identified metastasis mainly based on viable tumor cells instead of necrotic debris (**e**–**h**).

Using CAM, the key morphologic features for identification of metastatic colorectal cancer can be highlighted. An example of WSI with metastatic classical adenocarcinoma in the slide-level testing set is shown in Fig. 4a–d. The key morphologic features (highlighted with red color) for cancer identification were the areas of tumor cells but not the intervening stroma or inflammatory cells. Another example of metastatic classical adenocarcinoma with extensive tumor necrosis is shown in Fig. 4e–h. Our model identified this WSI as positive based on the areas of viable tumor cells rather than the necrotic debris.

**Fig. 5:**
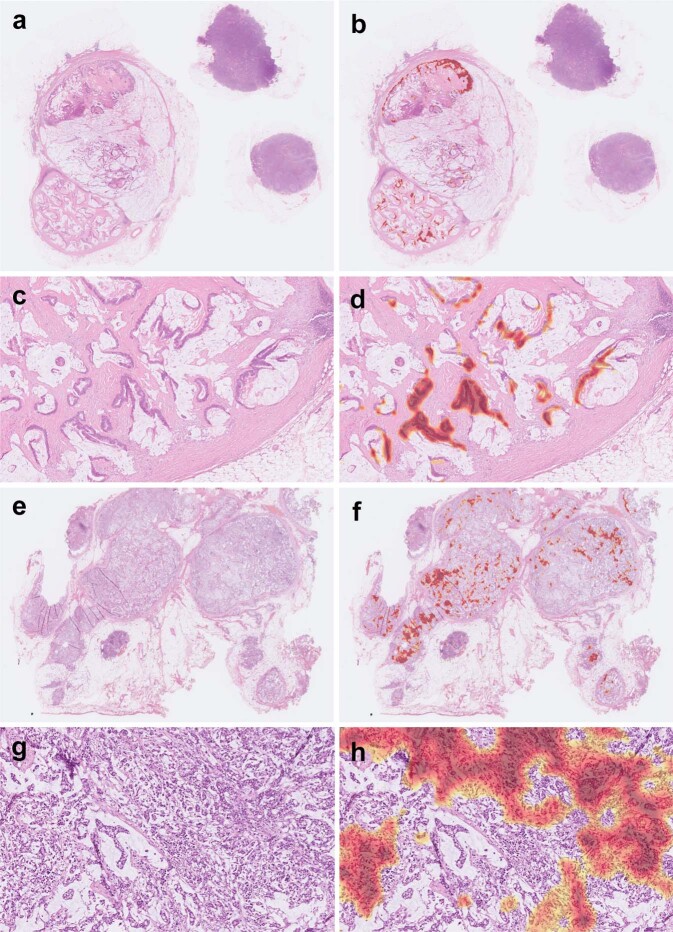
Examples of mucinous adenocarcinoma and signet ring cell/poorly differentiated adenocarcinoma in our slide-level testing set. An example of mucinous adenocarcinoma showed that our algorithm identified metastasis mainly based on viable tumor cells instead of mucin pools (**a**–**d**). An example of signet ring cell/poorly differentiated adenocarcinoma demonstrated that our algorithm identified metastasis mainly based on areas with higher density of tumor cells (**e**–**h**). Note that the areas with lower density of tumor cells were not highlighted.

An example of WSI with metastatic mucinous adenocarcinoma in the slide-level testing set is shown in Fig. 5a–d. The key morphologic features for identification were the areas of tumor cells but not the mucin pools. An example of signet ring cell/poorly differentiated adenocarcinoma is shown in Fig. 5e–h. Our model identified this WSI as positive mainly based on areas with higher density of tumor cells. Note that the areas with lower density of tumor cells were not highlighted. This could explain the slightly worse performance of our model in identifying signet ring cell/poorly differentiated adenocarcinoma.

**Fig. 6:**
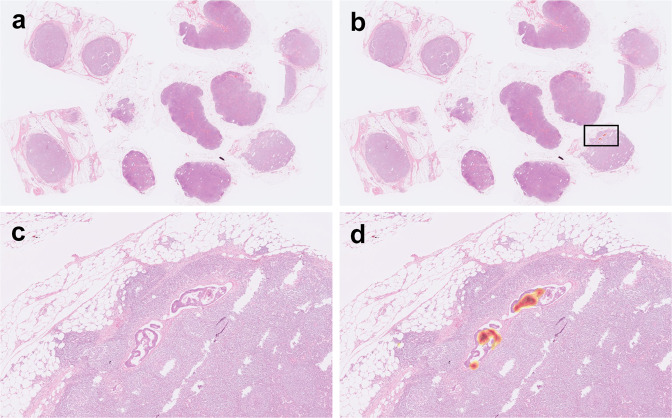
An example of micrometastasis in our slide-level testing set. Using class activation mapping, areas of the original whole-slide image (**a**) relatively important for positive prediction were highlighted with red color (**b**). A close-up view (**c**) (the square in **b**) and its highlighted areas (**d**) showed that our algorithm identified metastasis based on the small areas of tumor cells.

**Fig. 7:**
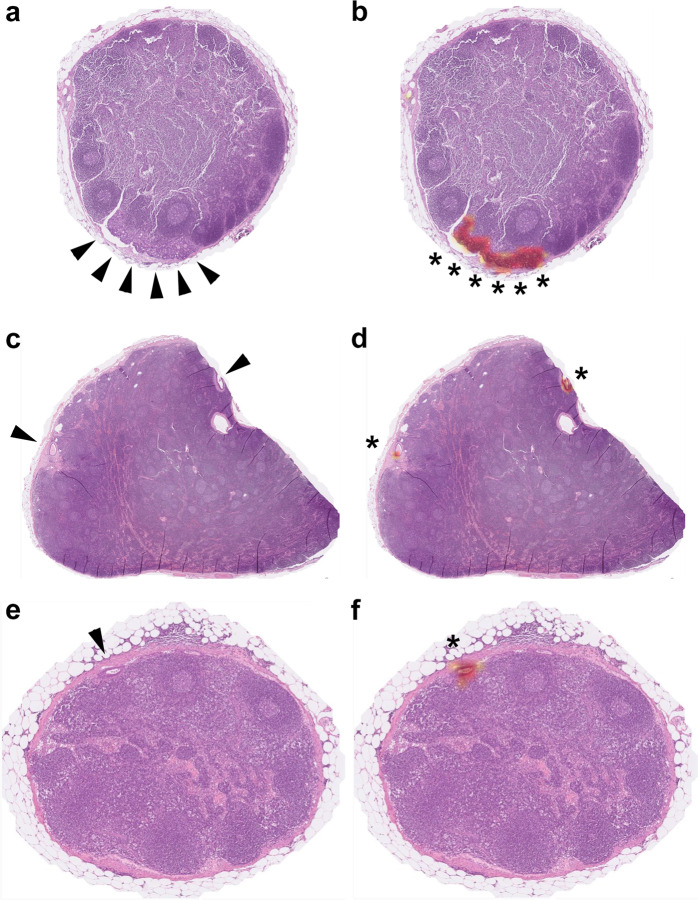
Examples of micrometastasis in our single-lymph node-level testing set. Using class activation mapping, areas of the original single-lymph node image (**a**, **c**, **e**) relatively important for positive prediction were highlighted with red color (**b**, **d**, **f**). Note that the areas important for identifying micrometastasis (asterisks) were the small areas of tumor cells (arrowheads).

Figure 6 shows an example of WSI with micrometastasis in our slide-level testing set. Note that multiple benign lymph nodes are present in this WSI. Our model successfully identified this WSI as positive based on the small areas of tumor cells. Figure 7 shows some single-lymph nodes with micrometastasis in our lymph node-level testing set. Our model also successfully identified these single-lymph nodes as positive based on the small foci of metastasis.

### Model performance on cases with preoperative chemotherapy

**Fig. 8:**
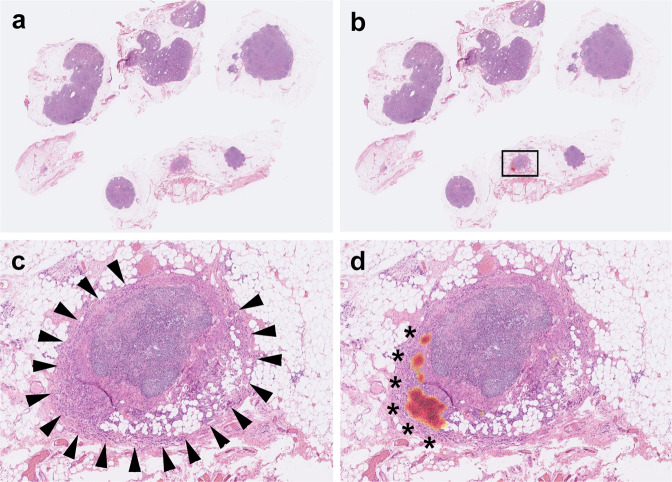
A false negative example of metastasis in a patient with preoperative chemotherapy. This whole-slide image (**a**) (close-up view: **c**) with a small focus (2.16 mm in size) of metastatic signet ring cell/poorly differentiated adenocarcinoma (arrowheads) was misclassified as negative. However, class activation mapping (**b**, **d**) still highlighted areas with relatively high density of tumor cells.

In our slide-level testing set, 59 WSIs were from patients with preoperative chemotherapy or concurrent chemoradiotherapy. Using a prediction threshold of 0.5, our algorithm achieved an accuracy of 98.31%. All six cases of micrometastasis were successfully detected, and no false positive cases were found. The only false negative case was a metastatic signet ring cell/poorly differentiated adenocarcinoma with a lesion size of 2.16 mm and low tumor cell density (Fig. 8a, c). Although our model falsely predicted this WSI as negative, the CAM still highlighted areas with relatively higher density of tumor cells (Fig. 8 b, d).

In our single-lymph node-level testing set, 28 lymph nodes were from patients with preoperative chemotherapy or concurrent chemoradiotherapy. Using a prediction threshold of 0.3, our algorithm achieved an accuracy of 96.43%. Both lymph nodes with micrometastasis were successfully detected, and there were no false positive cases. The only false negative case was a lymph node with isolated tumor cells.

## Discussion

The microscopic images of pathology slides are much more complicated than other types of medical images. Due to the large size (a few gigabytes on average) of high-resolution pathology WSIs, it is technically difficult to train a deep learning model directly using WSIs. Previously, most studies adopted a patch-based approach to perform deep learning on pathology images. Such an approach requires manual annotation, namely pixel-wise manual contouring of regions of interest (such as tumor areas) by experienced pathologists. The annotated regions are sliced into small patches, which are much more feasible for deep learning. This method has been successful in detecting [14–17], classifying [18–20], and grading [21, 22] tumors.

Of note, algorithms developed with patch-based deep learning methods performed well in detecting metastatic breast cancer in sentinel lymph nodes [5]. Their top-performing algorithm achieved an AUC of 0.994 for whole-slide prediction. Regarding detection of micrometastasis, their top ten algorithms had a mean AUC of 0.885 (range: 0.812–0.997), which was better than that of the best pathologist with time constraint (AUC = 0.808). A later study showed that such an algorithm can be used as an assisting tool for pathologists to detect micrometastasis [6]. With the assistance of the algorithm, the sensitivity of detecting micrometastasis by pathologists was increased significantly from 83 to 91% (*P* = 0.02). The average review time per image can be reduced for both micrometastasis (116–61 s; *P* = 0.002) and negative cases (137–111 s; *P* = 0.018). Their result showed the benefit of using a deep learning algorithm as an assisting tool to detect micrometastasis for pathologists.

Later on, a method of MIL was used to train neural networks with annotation-free WSIs [7]. Briefly, this method trains an AI model with a two-stage approach. At the first stage, WSIs are serially sliced into patches. The image patches are processed by a CNN image classifier. A certain number of image patches with the highest probability scores from a positive slide (or from a negative slide) are used as positive patches (or negative patches) in a training process to update the classifier. At the second stage, an additional slide-level classifier is trained to globally aggregate the patch-level results of all patches from a slide. They trained a deep learning model to detect metastatic breast cancer using more than 6500 WSIs of axillary lymph nodes. Their algorithm performed well with an AUC of 0.966. In the 403 positive cases of their testing set, 23 (5.7%) were misclassified as negative. Among the 23 false negative cases, 8 (34.8%) were macrometastasis, 13 (56.5%) were micrometastasis, and 2 (8.7%) were isolated tumor cells. Since micrometastasis is much less common than macrometastasis in practice, one can assume that their model performed worse in detecting micrometastasis. However, the exact performance of their algorithm in detecting micrometastasis was not described.

Recently, we developed a new method to train deep learning models using annotation-free WSIs with a slide-level label [8]. Unlike the MIL method which needs a slicing procedure and a two-stage training, our new method is a direct end-to-end training process using undivided WSIs. Our new method performed better than MIL in classifying lung cancer (AUC: 0.9594 vs. 0.9188 for adenocarcinoma; 0.9414 vs. 0.9032 for squamous cell carcinoma) [8]. In the present study, we used this new method to train an AI model using 1963 WSIs of lymph nodes. Our algorithm performed well in identifying metastatic colorectal cancer with an AUC of 0.9967. Compared to the study detecting metastatic breast cancer in axillary lymph nodes using MIL [7], we trained an AI model to detect metastatic colorectal cancer using our new method with less training data (1963 vs. >6500 WSIs) and better performance (AUC: 0.9967 vs. 0.966). In addition, our algorithm performed well in detecting both macrometastasis (AUC = 0.9993) and micrometastasis (AUC = 0.9956).

Detection of isolated tumor cells in H&E slides is extremely challenging for pathologists. Immunohistochemical study for cytokeratin is usually needed to detect all foci of isolated tumor cells [2, 4]. Since immunohistochemical study was not performed in this study, some lymph nodes with isolated tumor cells might have been labeled negative. This could partly explain the worse performance of our model in detecting isolated tumor cells. However, since a lymph node with isolated tumor cells is considered a negative node in the AJCC staging [3], detecting isolated tumor cells is of less significance than detecting micrometastasis.

Regarding different histologic subtypes, our algorithm performed slightly worse in detecting signet ring cell/poorly differentiated adenocarcinoma. This subtype of colorectal cancer is uncommon and has the greatest morphologic deviation from classical adenocarcinoma. The slightly worse performance of our model could be due to underrepresentation of such cases in the training set. In addition, our model is unlikely to achieve good performance for rare subtypes (such as small cell neuroendocrine carcinoma) of colorectal cancer absent in the training set.

Preoperative chemotherapy did not influence much the performance of our model. Although a WSI with a small focus (2.16 mm in size) of metastatic signet ring cell/poorly differentiated adenocarcinoma was missed by our model, the heatmap of CAM still highlighted areas with higher density of tumor cells (Fig. 8).

In practice, our AI model can be used as an assisting tool for pathologic diagnosis. A heatmap produced by CAM can highlight areas with high probability of metastatic tumor cells. Combining with an additional AI model for lymph node detection/segmentation, automated counting of positive/total lymph nodes could be achieved. However, such an automated counting system should only be used as an assistance to human counting, since a WSI might contain multiple slices of the same lymph node due to gross sampling.

In conclusion, here we develop for the first time a deep learning algorithm to detect nodal metastasis in colorectal cancer using undivided, annotation-free WSIs. Our model performed well in detecting both macrometastasis and micrometastasis. With no need of time-consuming manual annotation, our approach could accelerate development of new high-performance deep learning algorithms for pathology in the future.

## Data Availability

All data generated or analyzed during this study are included in this published article.
